# Improvement in liver function and gross motor function after umbilical cord-derived mesenchymal stromal cell transplantation in a child with autoimmune hepatitis and concurrent cerebral palsy: a case report

**DOI:** 10.3389/fmed.2026.1844700

**Published:** 2026-08-28

**Authors:** Liem Thanh Nguyen, Huong Thu Le, Doan Van Ngo, Thi Kieu Trang Phan

**Affiliations:** 1 Vinmec Research Institute of Stem Cell and Gene Technology, VinUniversity, Hanoi, Vietnam; 2 Regenerative Medicine and Cell Therapy Center, Vinmec Healthcare System, Hanoi, Vietnam; 3 Vinmec Smart City International Hospital, Hanoi, Vietnam; 4 Vinmec Times City International Hospital, Hanoi, Vietnam; 5 Innovation Health Sciences, VinUniversity, Hanoi, Vietnam

**Keywords:** autoimmune hepatitis, cerebral palsy, immunomodulation, liver function, mesenchymal stromal cells

## Abstract

Autoimmune hepatitis (AIH) is a complex immune-mediated liver disease of unknown origin that can rapidly progress to liver cirrhosis and end-stage liver failure. Standard treatment regimens typically involve immunosuppressive therapies, with most patients requiring lifelong maintenance. Recently, cell therapy has emerged as a potential therapeutic option for AIH. In this study, we report the case of a 2-year-old boy with type 2 autoimmune hepatitis and postcardiac arrest cerebral palsy who experienced clinical and biochemical improvement following umbilical cord-derived mesenchymal stromal cell (UC-MSC) infusion. Six months after the cell infusion, liver enzyme levels markedly reduced and remained stable throughout the 1-year follow-up period. At 1 year post-infusion, the patient demonstrated significant improvements in motor function, language skill, along with reduced hypertonia. At the most recent follow-up, his liver enzyme levels were within the normal range, and he had made substantial progress toward achieving developmental milestones. These clinical and biochemical changes suggest that UC-MSC therapy has potential as an adjunctive therapeutic approach. However, because this is a single, uncontrolled observation with several concurrent confounders, the clinical efficacy of UC-MSC therapy cannot be established from this case alone. Larger-scale studies with appropriate control groups, longer follow-up periods, and more detailed mechanistic assessments are required to determine whether UC-MSC therapy provides a true therapeutic benefit. No infusion-related adverse events were observed during follow-up.

## 1 Introduction

Autoimmune hepatitis (AIH) is a rare but severe chronic inflammatory liver disease with an unclear etiology that is thought to arise from a combination of genetic predisposition and environmental triggers. It is characterized by a dysregulated immune response targeting liver antigens, resulting in progressive hepatocyte injury and inflammation. If left untreated, AIH can progress to liver cirrhosis, hepatocellular carcinoma, or end-stage liver failure, necessitating urgent interventions (1). AIH affects people of all ethnicities worldwide with a female predominance, its epidemiological footprint varies significantly across age groups. In adults, the prevalence ranges from 10 to 25 per 100,000 individuals, whereas the pediatric incidence is markedly lower at approximately 0.2–0.4 per 100,000 children annually (2, 3). Crucially, compared with its adult-onset counterpart, pediatric-onset AIH often presents a more aggressive clinical phenotype and accelerates toward cirrhosis more rapidly (4).

Immunosuppressive treatment such as azathioprine combined with corticosteroids, has been the main treatment for AIH (5, 6). While effective at reducing liver inflammation and preventing disease progression, these medications carry the risk of severe adverse effects, including osteoporosis, diabetes, hypertonia, and immunosuppression (7, 8). Moreover, a significant proportion of patients relapse upon tapering or discontinuation of corticosteroids, necessitating long-term maintenance therapy (9, 10). Alarmingly, 10–20% of AIH patients demonstrate refractory disease, are unresponsive to standard treatment (9), and progress to liver failure, for which liver transplantation remains the only definitive option (11).

Emerging regenerative therapies, such as mesenchymal stromal cells (MSCs), offer a promising alternative for managing refractory AIH. MSCs exert pronounced immunomodulatory effects by modulating T-cell responses, shifting macrophage polarization and reducing inflammatory cytokines, as well as antifibrotic and trophic (paracrine) properties that may mitigate liver injury (12, 13). These unique immunoregulatory properties, rather than direct transdifferentiation into hepatocytes, underpin their potential as a therapeutic option for autoimmune and inflammatory liver diseases (14). However, clinical evidence supporting their use in AIH remains scarce.

In this report, we describe the first case of a 2-year-old boy with a severe course of type 2 AIH, who subsequently developed cerebral palsy (CP) at 12 months due to cardiac arrest. The patient received allogeneic UC-MSC infusion, resulting in a remarkable clinical response. This case highlights the potential of regenerative medicine to address unmet needs in the treatment of refractory autoimmune liver diseases.

## 2 Case presentation

**Figure 1:**
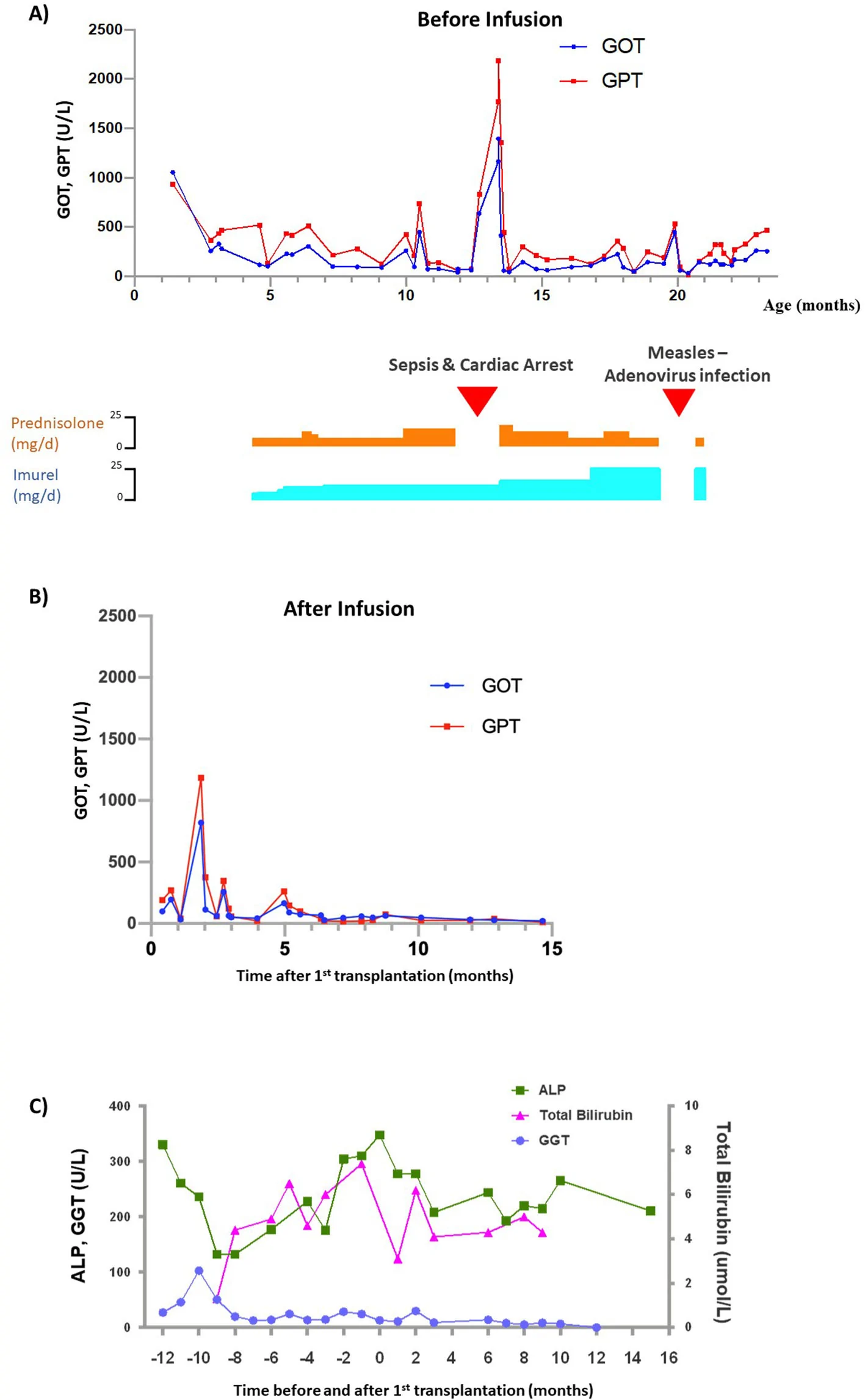
Timeline of liver function testing, symptom presentation, and treatment **(A)**. Influence of allogeneic mesenchymal stem cell transplantation (MSCT) therapy on serum GOT, GPT, GGT, ALP and bilirubin **(B, C)**.

A boy was born in September 2017 by C-section at 38 weeks of gestation (birth weight 3,350 g), with no perinatal asphyxia or jaundice, and developed diarrhea and a purpuric rash at 15 days of age. Examination revealed that the liver and spleen were palpable 2 cm below the costal margins. He received antibiotics and intravenous fluids for 20 days. At 1 month and 12 days, he presented with hematemesis and subcutaneous bleeding. Laboratory tests revealed a platelet count of 6 G/L, a GOT concentration of 1,056 U/L, a GPT concentration of 936 U/L and a cytomegalovirus (CMV) DNA load of 7.84 x 10^5^ copies/mL. He was diagnosed with thrombocytopenic purpura, hepatitis, and CMV infection, and treated with intravenous ganciclovir for 21 days. The platelet count (158 G/L) and transaminase normalized, and the CMV DNA load decreased to 3,600 copies/ml. Two weeks after discharge, the concentration of GOT and GPT increased to 260 U/L and 367 U/L, respectively, and oral L-arginine (1,000 mg/day) was started; 2 weeks later, the concentrations were 330 U/L and 434 U/L, respectively, and Carduus marianus 100 mg/day (silymarin 70 mg and silybin 30 mg) and vitamin E were added. At 4.5 months, anti-liver-kidney microsome type 1 (anti-LKM-1) was positive (36.2 IU/mL), antinuclear antibody was borderline (1.2 OD) and anti-mitochondrial M2 antibody was equivocal (13.8 AU/mL); anti-liver cytosol type 1, anti-Sm, anti-asialoglycoprotein receptor, MPO-ANCA and PR3-ANCA were negative. Serum IgG concentration at approximately 6 months was 6.96 g/L (age-appropriate). Hepatitis A, B and C serology were negative, and serum alpha-1-antitrypsin concentration was normal (1.33 g/L). Type 2 autoimmune hepatitis was diagnosed and immunosuppression started with prednisolone (10 mg/day) and azathioprine (Imurel, 10 mg/day). After 2 months, biochemical remission was not achieved (GOT 305 U/L and GPT 512 U/L). Therapy was interrupted twice: from September to November 2018 (sepsis and cardiac arrest) and from May to September 2019 (measles-adenovirus coinfection). Both drugs were withheld during these episodes; Imurel was resumed at 20 mg/day after approximately month 25 (Figure 1A).

In September 2018, at 12 months of age, the patient presented with fever, lethargy, diarrhea, and frequent vomiting, and deteriorated to multiorgan dysfunction syndrome and cardiac arrest within 48 h. He was diagnosed with sepsis and managed with intensive care with seven days of mechanical ventilation, 5 days of oxygen support, and dialysis; treatment included meropenem, tobramycin, fluconazole, metronidazole, and intravenous immunoglobulin. GOT and GPT peaked at 1,398 U/L and 2,185.4 U/L, respectively. He was diagnosed with hypertonia, motor disability, agnosia, twisting movements, and cerebral palsy (CP). Brain MRI revealed enlargement of the bifrontal subarachnoid space.

The liver injury in this patient had more than one contributing mechanism, which should be considered separately. Neonatal episode of severe thrombocytopenia, very high transaminases and a high CMV DNA load that fell after ganciclovir was most consistent with CMV-associated hepatitis. The extreme increase in the GPT (up to 2,185.4 U/L) during later septic episodes with multiorgan dysfunction and cardiac arrest is consistent with sepsis-associated and hypoxic/ischemic (“shock liver”) injury rather than autoimmune activity. Drug-induced liver injury cannot be fully excluded. The diagnosis of AIH rests on persistent transaminase elevation with strongly positive anti–LKM-1 that continued after CMV clearance and resolution of systemic insults, together with the response to immunosuppression.

## 3 Therapeutic intervention

### 3.1 UC-MSC preparation and characterization

The MSC line used in this case was selected from the MSC biobank, which was characterized according to the ISCT guidelines, and was cultured under xeno-free and serum-free conditions as previously described (15). Cells were isolated from the single umbilical cords of healthy newborns whose mothers had a full-term pregnancy and who tested negative for infections, including HIV, cytomegalovirus (CMV), Epstein-Barr virus (EBV), hepatitis A (HAV), hepatitis B (HBV), Hepatitis C (HCV), syphilis, and chlamydia. After collection, the umbilical cords were immediately transferred to the Stem Cell Technology Research Laboratory at Vinmec Times City Hospital, which operates under ISO 14644–1 standards, for processing and MSC isolation. The cells were cultured in a serum-free and animal component-free environment, as previously described (15). The MSCs were maintained at 37 °C, under 5% CO_2_ and 95% humidity. Once they reached 80% confluency at passage 1 (P1) to P3, the cells were harvested and characterized. These included assessments of surface markers, karyotype stability and purity. Only the MSCs that met all the quality characterization and sterility criteria were cryopreserved in an automated Brooks system at −196 °C, to form a master cell bank.

To prepare cells for therapy, UC-MSCs are thawed from P3 and cultured under xeno- and serum-free conditions, without the use of antibiotics. Upon achieving a confluency of 80% at P5, the UC-MSC are harvested and evaluated against established quality criteria. These assessments include cell count, viability, expression of MSC markers (according to ISCT standards), sterility (ensuring the absence of bacteria, fungi, and mycoplasma), and endotoxin levels (maintained within the limit of 5 EU/kg/hour) described in Table 1. After meeting the release criteria, the cells are suspended in 0.9% NaCl physiological saline at the required density for infusion, targeting 1 × 10^6^ cells/kg of body weight, with a viability of ≥ 90%. Both infusions given to this patient were prepared from the same single-donor line.

**Table 1:** Quality control of UC-MSC products for transplantation.

Quality control parameter	The first treatment	The second treatment
Cell passage	5	5
Dose	1x10^6^ cells/kg	1x10^6^ cells/kg
Viability	94%	96.8%
MSC marker (CD105, CD90, CD73, Negative markers)	CD105: 99.83%, CD90: 99.99%, CD73: 99.99%, Negative markers: 1.18%	CD105: 97.65%, CD90: 99.77%, CD73: 99.99%, Negative markers: 0.1%
Bacteria and fungi	Negative	Negative
Mycoplasma	Negative	Negative
Endotoxin	< 0.05 EU/mL	< 0.05 EU/mL

### 3.2 Cell infusion

After informed consent was obtained from the patient's family and approval from the hospital's Board of Directors, UC-MSC therapy was initiated. The patient received two separate infusions of allogeneic UC-MSCs from the same single donor line; the two doses were administered 21 months apart. The first infusion (1x10^6^ cells/kg in 10 mL of 0.9% NaCl) was given intravenously on 11 September 2019, at 23 months of age. Standard treatment for AIH was continued after the first infusion. The second infusion (1 × 106 cells/kg) was given intrathecally at the L4–L5 interspace under general anesthesia on 8 June 2021, according to an established protocol, and was completed within 30 min (16).

### 3.3 Outcome measurement

Liver function was assessed by serum glutamic oxaloacetic transaminase (GOT), serum glutamic pyruvic transaminase (GPT), alkaline phosphatase (ALP), gamma-glutamyl transferase, bilirubin and albumin levels. Motor and developmental function were assessed with the Denver Developmental Screening Test II (DDST-II), the Gross Motor Function Measure (GMFM-88), the Gross Motor Function Classification System (GMFCS) and the Quality of Upper Extremity Skills Test (QUEST; dissociated movement and grasp subscales). Brain MRI was performed before and 21 months after the first infusion.

## 4 Results

### 4.1 Evaluation before cell therapy

At presentation to Vinmec on 29 August 2019 the patient was conscious and communicative, with hypertonia and poor motor function. He could roll, crawl and sit unsteadily, but could not pull to stand or walk. He could grasp large objects but could not perform a pincer grip or point. He produced no single words. He recognized familiar faces, understood spoken words and expressed emotions. Activities of daily living were caregiver dependent; chewing and spoon use were poor, and he did not signal toileting needs. Baseline laboratory values were as follows: white blood cells, 11.72 G/L; neutrophils, 32%; lymphocytes, 49.1%; platelets, 210 G/L; red blood cells, 5.61 T/L; hemoglobin, 136 g/L; MCV, 69.5 fL; MCHC, 349 g/L; prothrombin time, 84%; APTT, 36.3 s; fibrinogen concentration, 2.86 g/L; GOT, 255.4 U/L; GPT, 466.9 U/L; ALP, 348 U/L; CRP, 0.90 mg/L; and Na/K/Cl 139/4.6/111 mmol/L. Between diagnosis of AIH and the first infusion, the median GOT was 121 U/L and the median GPT was 240.7 U/L. The baseline GMFCS was level IV, the GMFM-88 was 54 and the QUEST was 55.55. On the DDST-II, personal–social and expressive language was at the 11-month level, receptive language was at 18 months, and fine and gross motor skill were at 6 months.

### 4.2 Cell therapy and improvement

Imurel was continued, and rehabilitation for CP was maintained throughout the follow-up period. The first infusion was given 19 months after the diagnosis of AIH and 11 months after the diagnosis of CP.

No infusion-related adverse events were recorded. Liver enzymes were measured every 2 weeks after the first cell infusion. At approximately day 58, the GOT and GPT increased to 820.6 U/L and 1,184 U/L, respectively (Figure 1B), whereas GGT (30 U/L), total bilirubin (6.2 μmol/L), albumin (42.3 g/L), the INR (1.15) and fibrinogen (2.46 g/L) remained normal. Transaminases then declined and returned to the reference range within approximately 6 months and remained stable thereafter (Figures 1A, B). Azathioprine was discontinued 10 months after the first infusion. ALP concentration decreased from 348 U/L at baseline to 214.8 U/L at 9 months and remained stable (Figure 1C). Bilirubin and gamma-glutamyl transferase levels remained within the reference range throughout follow-up.

At 21 months after the first infusion (age 44.5 months), hypertonia was reduced, and chorea and athetosis had lessened. He could sit up unaided, move from lying to sitting without support, and pull to stand while holding furniture, but could not walk. The grasp was secure with both hands, and the pincer grip improved. He could feed himself, brush his teeth, wash his face, remove his trousers, dress with partial support and signal toileting needs. He identified body parts, colors and pictures of animals; spoke many single words, understood a range of words and answered simple questions with gestures. The GMFM-88 increased from 54 to 125, the GMFCS improved to level III and the QUEST to 87.03. Further motor results are shown in Table 2, and developmental age-equivalent scores in Table 3. Motor and developmental assessments were performed at routine follow-up visits rather than at fixed intervals relative to the infusions.

**Table 2:** Changes in the GMFM88, GMFCS, and QUEST before and after MSC infusion.

Assessment	Before infusion	After 1^st^ infusion	After 2^nd^ infusion
**GMFCS**	**IV**	**III**	**III**
GMFM-88		
Lying and rolling	44	51	51
Sitting	7	45	60
Crawling and Kneeling	3	23	36
Standing	0	5	15
Walking, running, and jumping	0	1	12
Total	**54**	**125**	**174**
QUEST	55.55	87.03	92.59

The GMFM-88 values are presented as raw scores (maximum total score = 264). QUEST values are presented as percentage scores ranging from 0% to 100%.

**Table 3:** Developmental age-equivalent scores (months) measured using the Denver Developmental Screening Test II before and after UC-MSC administration.

DDST-II domain	DDST scale score		
	Before infusion	After 1^st^ infusion	After 2^nd^ infusion
Age at DDST	23.5	44.5	72
Personal-Social	11	15	22–23
Fine motor	6		24–26
Right hand	–	17	–
Left hand	–	11	–
Expressive language	11	20–22	30–33
Receptive language	18	44	72
Gross motor	6	10	13

DDST-II values represent developmental age-equivalent scores expressed in months rather than numerical test scores.

Three months after the second cell infusion (24 months after the first), motor function improved further. He could take steps while holding furniture and stand unsupported for short periods. He understood language well, spoke in longer sentences, answered appropriately, asked questions, and told simple stories. Serial results are shown in Table 2.

**Figure 2:**
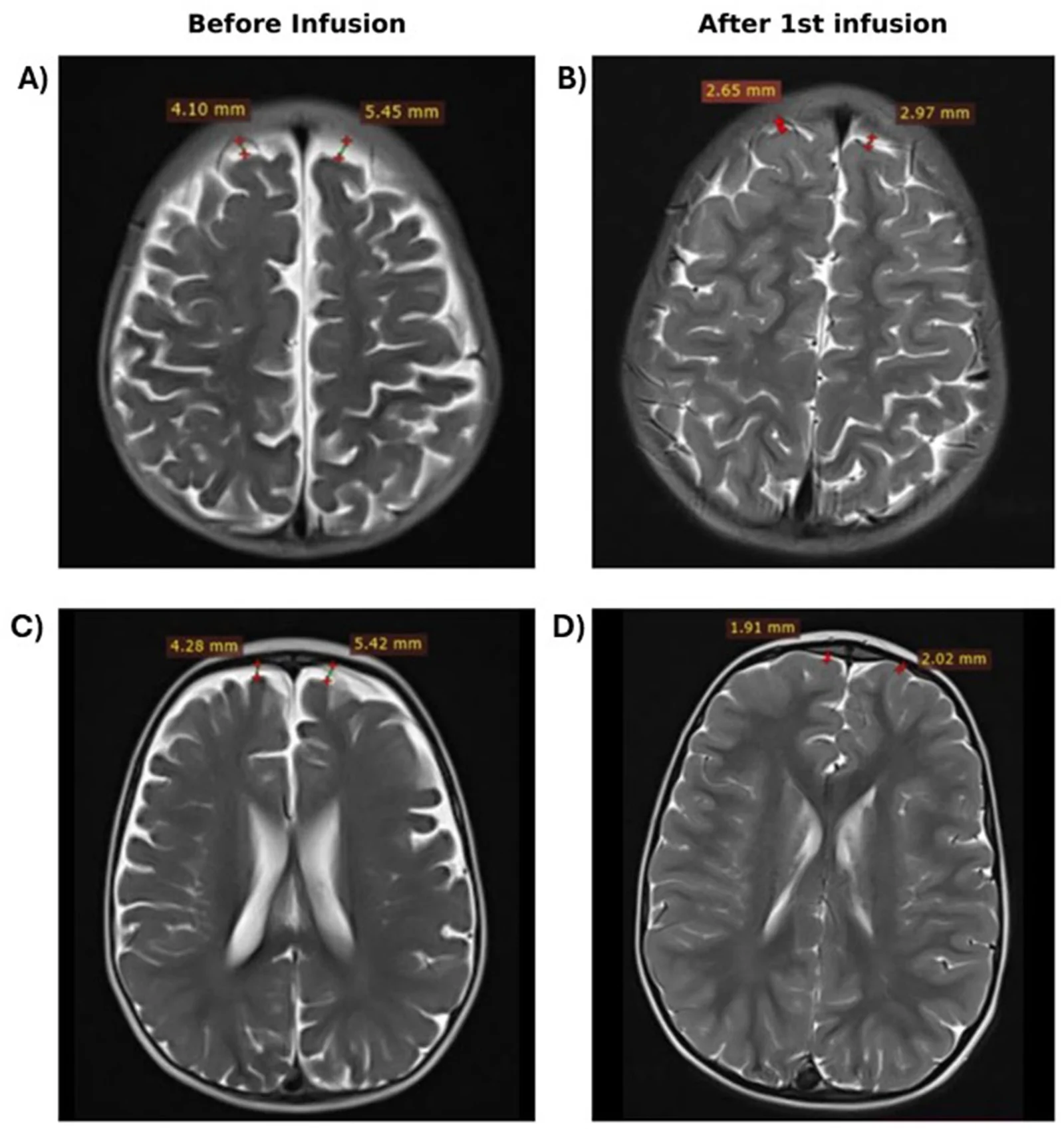
Axial T2-weighted magnetic resonance imaging (MRI) patterns in cerebral palsy following hypoxic–ischemic injury after cardiac arrest CP changes after cell therapy. **(A, C)** Axial T2W images obtained before cell infusion demonstrated widening of the superficial subarachnoid space (arrows). **(B, D)** This space was visibly reduced 21 months after the initial cell infusion.

On a brain MRI performed 2 months after the diagnosis of CP, the bifrontal subarachnoid space measured 4.10–5.45 mm (Figures 2A, C). At 21 months after the first infusion, this extra-axial space was reduced to 1.91–2.97 mm at comparable axial slice levels on the same T2-weighted sequence (Figures 2B, D).

## 5 Discussion

In this single, uncontrolled case, clinical and biochemical improvement was observed temporally after UC-MSC administration in a child with type 2 AIH complicated by CP. This dual pathology presents a unique therapeutic challenge, as AIH requires immunosuppressive treatment, while CP needs rehabilitation for motor and cognitive impairments. UC-MSC therapy appeared to provide benefits for both conditions, with no adverse effects observed during follow-up.

Serum transaminases decreased after UC-MSC administration and remained within the reference range thereafter, and Imurel was withdrawn 10 months after the first infusion. The transient increase in the GOT and GPT on day 58 was not accompanied by any change in synthetic function or cholestatic indices, indicating that it did not represent clinically significant hepatic decompensation. Similar transient biochemical flares have been reported after cell therapy and have been attributed to short-lived immune activation or to remodeling and repair within the liver microenvironment (17, 18), although their clinical significance remains uncertain. MSCs are known to modulate T-cell responses, shift macrophage polarization, reduce fibrosis and support hepatocyte regeneration (19–23), and these properties provide a plausible basis for the observed course (21–23). However, direct evidence that MSC administration was responsible for the clinical outcomes observed in this patient is lacking.

Gross and fine motor function, cognition and language improved after both infusions, with gains in GMFM-88, GMFCS, QUEST and DDST-II scores and a reduction in hypertonia, and with increasing independence in feeding and dressing. MSC therapy has been reported to attenuate neurological deficits by modulating inflammation, promoting neural repair and the release of neurotrophic factors (24, 25). The second infusion was given intrathecally, a route that bypasses the blood-brain barrier and allows cells more direct access to injured neural tissue, and which has been associated with better functional outcomes than systemic administration in neurological disease (25, 26). The relative contribution of the two routes cannot be determined from a single case (25).

The bifrontal subarachnoid space was narrower at 21 months than at baseline. This may reflect a reduction in chronic inflammation or ongoing structural maturation, but in the absence of a control it cannot be attributed to cell therapy, and normal brain growth in a child of this age is an adequate alternative explanation.

This report has important limitations. It is a single, uncontrolled observation, which precludes causal inference and generalization. Several competing causes of liver injury, including CMV hepatitis, sepsis-associated and hypoxic-ischemic injury, and drug-induced liver injury cannot be fully excluded; the neurological picture followed hypoxic-ischemic injury after cardiac arrest. Continued immunosuppression, intensive rehabilitation and normal childhood development are major confounders for both hepatic and neurodevelopmental gains, and the mechanisms proposed above are speculative. No mechanistic or immunological assays were performed.

In conclusion, clinical, biochemical and functional improvements were observed during long-term follow-up after two separate infusions of allogeneic UC-MSCs in a child with type 2 AIH and CP, with no adverse infusion-related events. The contributions of concomitant treatment, rehabilitation and natural disease evolution cannot be excluded. Controlled studies with predefined outcomes and longer follow-up and mechanistic assessments are required to determine the safety, feasibility and therapeutic role of UC-MSCs under these conditions

## Data Availability

The original contributions presented in the study are included in the article/supplementary material, further inquiries can be directed to the corresponding author.
